# Scedosporium Sinusitis: A Rare Opportunistic Infection

**DOI:** 10.7759/cureus.43475

**Published:** 2023-08-14

**Authors:** Saipriya Ayyar, Rebekah Lantz, Asif Khan

**Affiliations:** 1 Boonshoft School of Medicine, Wright State University, Dayton, USA; 2 Department of Internal Medicine, Miami Valley Hospital, Dayton, USA; 3 Department of Infectious Disease, Dartmouth Hitchcock Medical Center, Lebanon, USA

**Keywords:** maculopapular rash, human immunodeficiency viruses (hiv), infectious disease pathology, ent procedures, scedosporium, hemophagocytic lymphohistiocytosis (hlh), invasive fungal sinusitis, infections in neutropenic fever, opportunistic fungal infection, scedosporium sinusitis

## Abstract

*Scedosporium* sinusitis is an opportunistic fungal infection that is difficult to treat due to its inherent resistance to many antifungal agents. Infections may cause both localized or disseminated disease usually in skin and soft tissues. Immunocompetent persons are typically unaffected and disseminated disease occurs in immunocompromised hosts. *Scedosporium*is a common hyaline mold causing sinopulmonary disease in those with hematologic malignancies and neutropenia. A 38-year-old Caucasian male with a medical history significant for HIV with intermittent treatment compliance, high-grade diffuse large B cell lymphoma (DLBCL) on chemotherapy, and hemophagocytic lymphohistiocytosis (HLH) presented with right-sided facial pain and fever. Maxillofacial computed tomography (CT) showed thickening and opacification of the sphenoid and maxillary sinuses concerning for fungal sinusitis. Endoscopic transsphenoidal debridement showed fungal growth of *Scedosporium* and the patient’s blood cultures were ultimately negative. The patient underwent debridement of fungal sinusitis as well as right medial maxillectomy and ethmoidectomy. A three-month course of voriconazole was started and completed with weekly liver enzyme tests to monitor medication side effects. He has since been observed well as an outpatient with his oncologist after three months loss to follow-up and his infection has resolved.

## Introduction

*Scedosporium* species are a type of fungus commonly found in stagnant and polluted water. They often cause sinopulmonary, central nervous system, and disseminated infections in patients who have had contact with spores, typically through inhalation or exposure to contaminated water. Localized infections often involve specific tissues such as the respiratory tract, skin, soft tissues, sinuses, nasal septum, and bone [1]. While the invasive form of infection is mostly limited to patients with hematologic malignancy or neutropenia, disseminated infections may also occur, especially in severely immunocompromised patients.

As a result, *Scedosporium* remains a common cause of fungal infections in immunocompromised patients including those with cystic fibrosis, human immunodeficiency virus (HIV), acquired immunodeficiency syndrome (AIDS), chronic granulomatous disease (CGD), solid organ transplant recipients, neutropenia, and hematologic malignancy [2]. The overall mortality is 46.9% and the mortality increases to 87.5% in cases of disseminated disease [3]. Furthermore, disseminated infections have a mortality of up to 70% in patients with hematologic malignancies compared to patients with other immunocompromising factors [4]. It is important to be cognizant of this infectious differential in at-risk patients.

In this report, we discuss the importance of considering *Scedosporium* as a differential etiology of infection and the prompt treatment of this life-threatening condition.

## Case presentation

**Figure 1:**
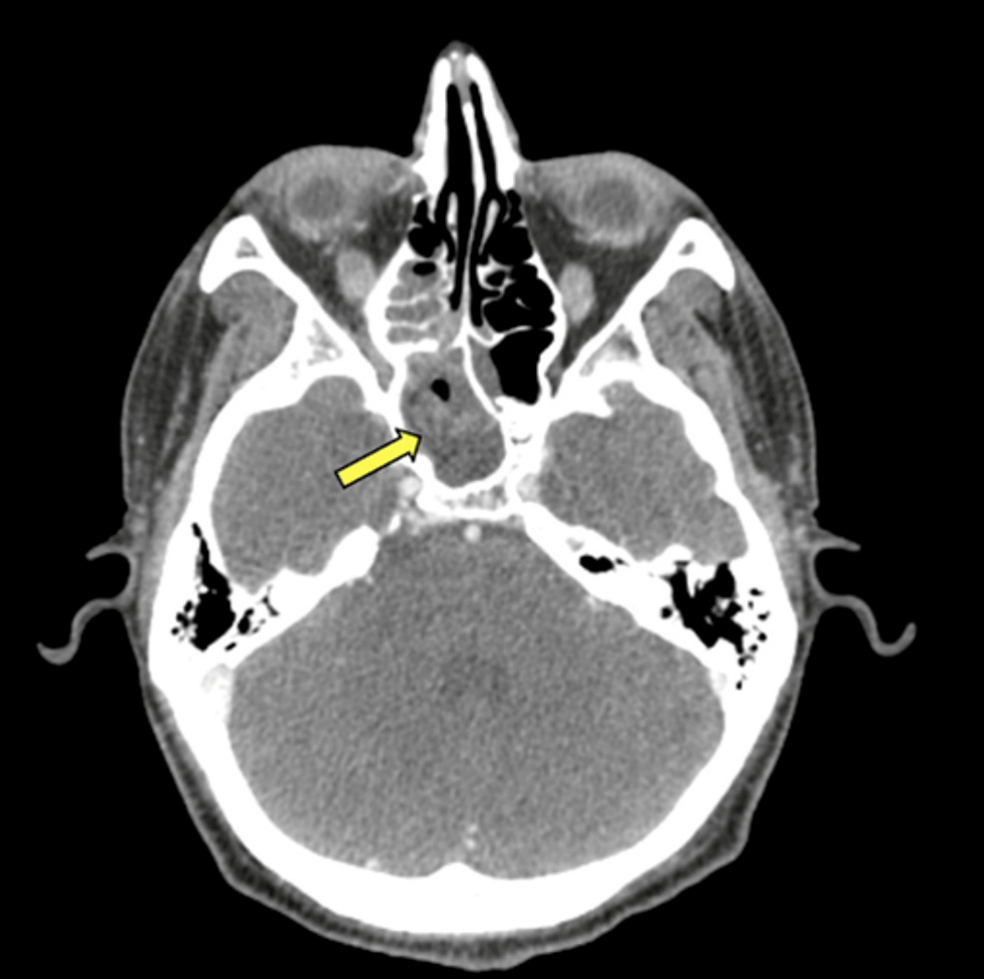
Initial CT maxillofacial with contrast Findings concerning for invasive fungal sinusitis with near complete opacification of the right sphenoid sinus (yellow arrow).

**Figure 2:**
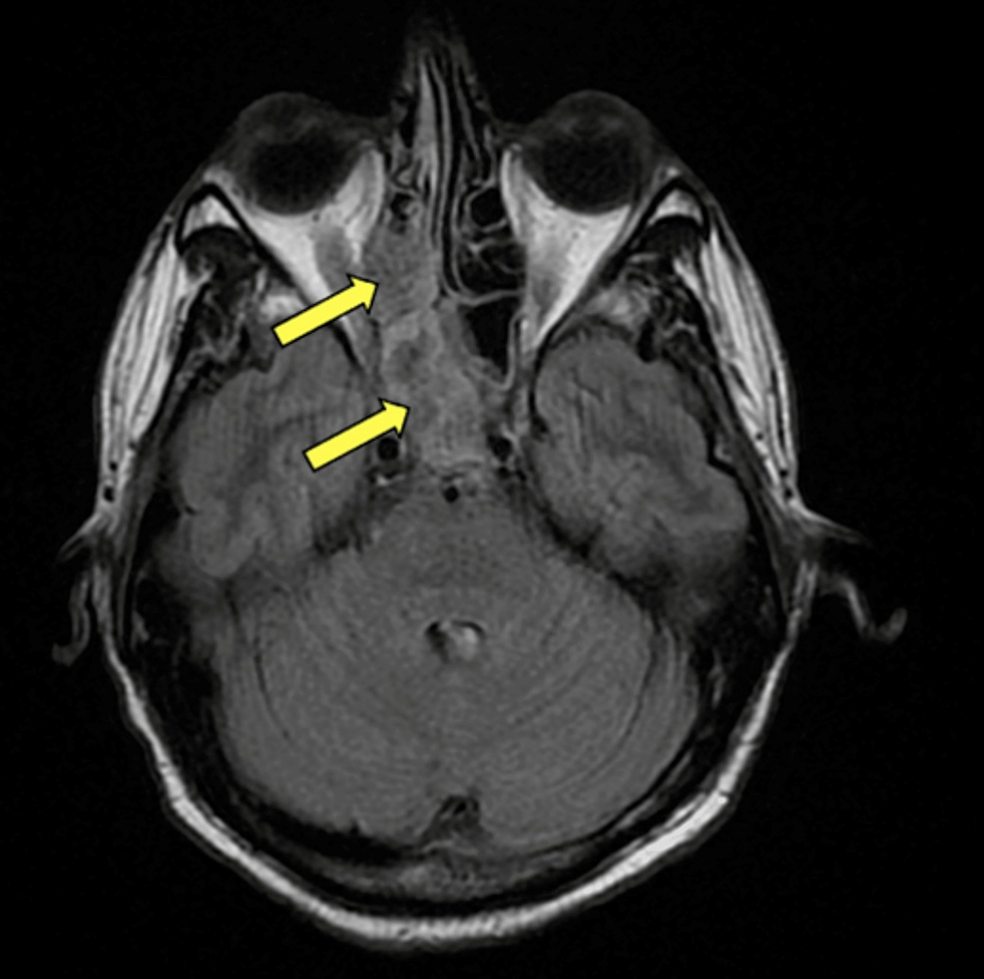
MRI orbit face and neck with contrast Findings show right sphenoethmoidal fungal sinusitis (yellow arrows). There is infection and an 8 mm sinonasal polyp in the right sphenoethmoidal recess/posterior superior nasal canal.

A 38-year-old Caucasian male with a medical history of HIV (CD4+ 81/uL) on antiviral treatment, diffuse large B-cell lymphoma (DLBCL) on chemotherapy, and hemophagocytic lymphohistiocytosis (HLH) presented to the emergency department due to right frontal headache, right-sided jaw pain, and fever. On arrival, the patient was found to be febrile with a temperature of 102.8°F and tachycardic in the low 100s. He did not have any focal neurologic signs or meningismus concerning for central nervous system (CNS) involvement. The presentation was initially attributed to oral thrush, fungal skin infection, and neutropenic fever. He had completed cycle 5 of pegfilgrastim just prior to admission. Empiric intravenous vancomycin, cefepime, and fluconazole were started for neutropenic fever and suspected fungal source. Computed tomography (CT) was obtained (Figure 1) followed by a more detailed magnetic resonance image (MRI) brain as well as an orbit face and neck with and without contrast (Figure 2).

**Figure 3:**
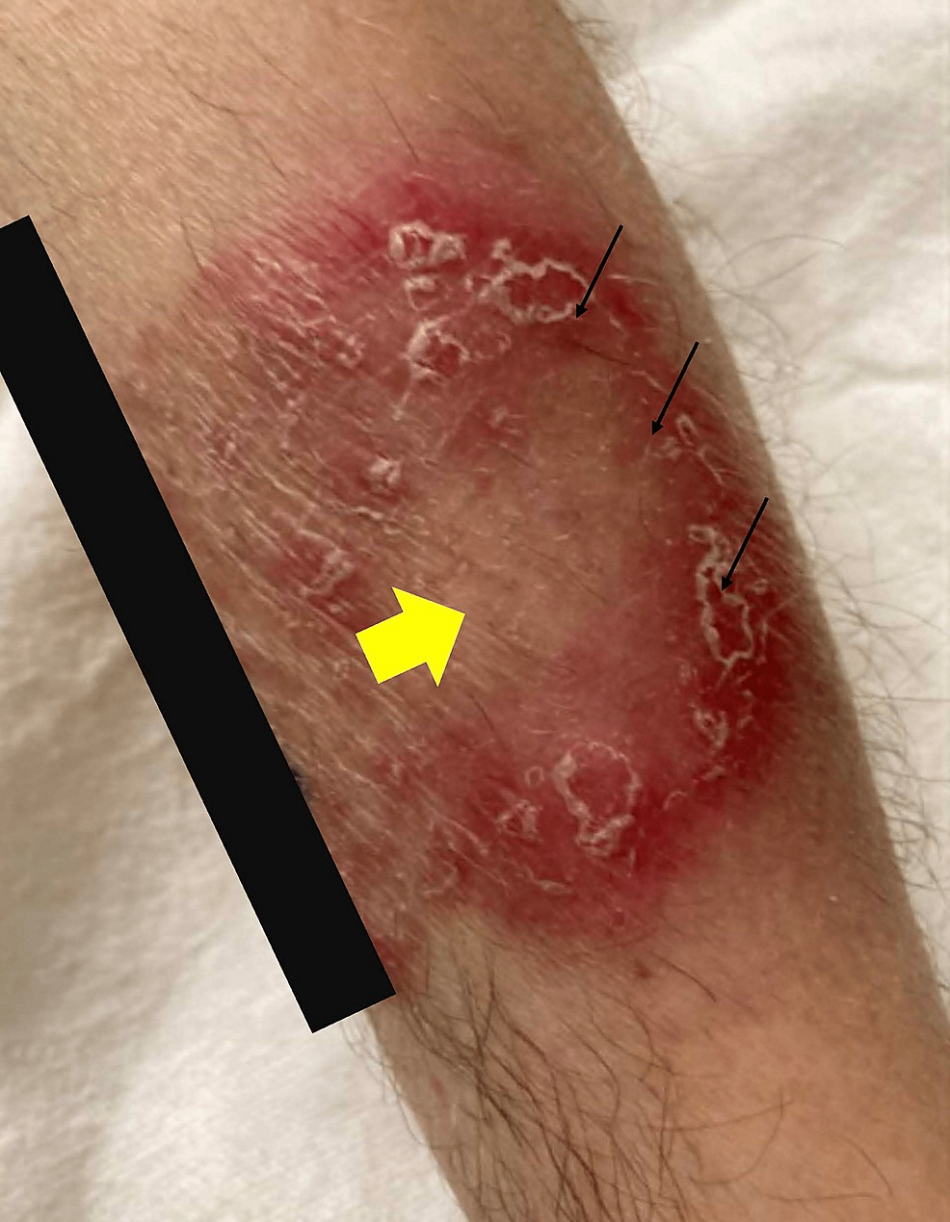
Maculopapular rash (black arrows) on presentation to emergency department with circular and serpiginous distribution and central clearing (yellow arrow) and scale on the right arm.

His prophylactic doses of sulfamethoxazole/trimethoprim and acyclovir were continued as well as IV fluconazole. An infectious disease specialist recommended a prolonged course of fluconazole swish and swallow for 10 months and topical triamcinolone as the patient was at risk of fungal infection, though a fixed drug reaction as a cyclical rash related to chemotherapy cycles was considered. The lesion was described as a maculopapular rash with circular and serpiginous distribution and central clearing with a scale on his arms and extremities (Figure 3). Dermatology suggested a diagnosis of Majocchi’s granuloma, with a plan to follow culture results and obtain an outpatient biopsy. The patient remained leukopenic and neutropenic through his hospital course.

**Figure 4:**
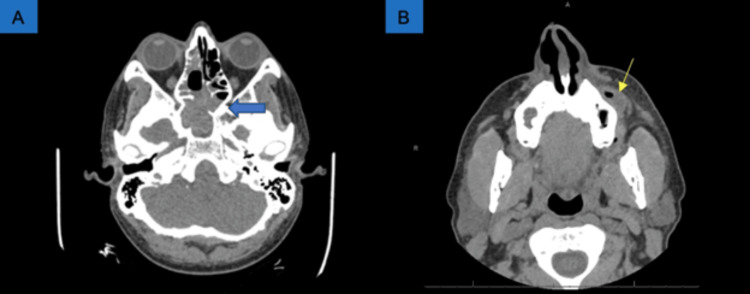
Repeat CT maxillofacial on day 7 of admission (A) Postsurgical changes with partial ethmoidectomy and sphenoidectomy. Soft tissue obstruction of contralateral left sphenoid sinus (blue arrow); (B) Abscess of #12 (yellow arrow is enhanced over radiologist’s indicator).

**Figure 5:**
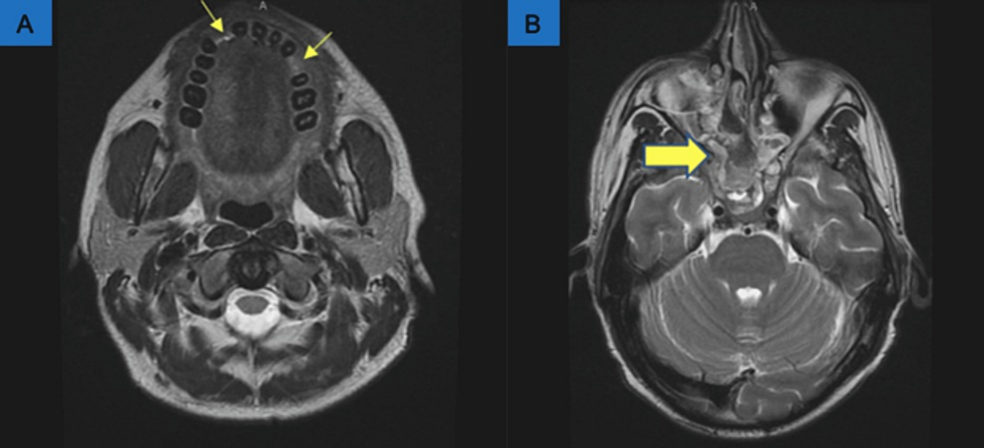
Repeat MRI orbit face and neck (without contrast) on day 8 of admission showing now absent dentition (thin yellow arrows) as well as postoperative changes (thick yellow arrow).

The otorhinolaryngologist, ear-nose-throat (ENT) provider, was consulted and a nasal debridement was done by endoscopy. Subsequent CT showed post-operative findings of bony osteotomy, partial ethmoidectomy, and bone resection of the sphenoid sinus drainage tract. There appeared to be soft tissue obstruction of the osteomeatal unit on the left but without gas. He had notable dental caries of #13 and adjacent soft tissue abscess. This was associated with gas and cellulitis (Figures 4-5). The dental specialist assessed the patient and #7 and #12 were intraorally incised and drained at bedside. The sinus findings were suggestive of persistent right sphenoethmoidal fungal sinusitis and ENT reoperated with stereotactic navigation and endoscopic endonasal expansion approach to the skull base with a similar postoperative diagnosis.

Blood cultures were ultimately negative including acid staining. Intraoperative cultures were consistent with *Streptococcus* and *Staphylococcus* flora on aerobic and anaerobic cultures. Right sphenoid culture grew *Scedosporium* species without determined sensitivities. Infectious Diseases recommended discontinuation of vancomycin after culture results. Cefepime was considered sufficient and discontinued after his 10-day hospitalization. Oral voriconazole was given for a three-month course.

## Discussion

*Scedosporium* remains a rare cause of infection in immunocompetent, immunocompromised, and immunosupressed states. It may have a wide range of manifestations from soft tissue to CNS infections with the most common sites being skin and soft tissue, bone, and the pulmonary tree. However, with increasing awareness, unique sites of *Scedosporium* inoculation have been noted. For example, a case series showed that pterygium surgery increased the risk of *Scedosporium* infection to the cornea and sclera [5]. An unusual case of intravenous catheter-related *Scedosporium* infection was evidenced by Eldin, et al [6]. In fact, the presentation of *Scedosporium* infection has been so varied that it has previously been mistaken for pyoderma gangrenosum [7].

Our patient had multiple immunocompromising factors which increased the risk of infection. In addition to the history of HIV, hematologic malignancy, and chemotherapy-inducing neutropenia seen in this case, *Scedosporium* infections also commonly occur in organ transplant recipients and cystic fibrosis patients [2,8,9]. Typically, infection can onset in HIV-positive patients when CD4+ cell counts are less than 100/uL, consistent with our case (CD4+ 81/uL), as well as CNS manifestations with CD4+ less than 50/uL [10]. A case of disseminated disease in a stem cell transplant recipient with a history of acute myeloid leukemia warns of the dangerous possibility of disseminated *Scedosporium* infection in patients with several risk factors [11]. Immunosuppressed patients on anti-rejection therapies or immunosuppressants such as steroids and biologics are at risk [12,13]. The complex medical history of our patient further demonstrates the need for increased vigilance in monitoring for *Scedosporium* infections in immunocompromised patients.

Despite being an opportunistic fungus, *Scedosporium* often causes infections in immunocompetent hosts. Many cases in immunocompetent hosts occur due to trauma [14.15], such as near-drowning experiences [1]. However, *Scedosporium* has also presented as a cause of osteomyelitis [15,16], brain abscess after extracorporeal membrane oxygenation (ECMO) [17], and endocarditis in immunocompetent states [18].

*Scedosporium* is an inherently multi-resistant species making it difficult to treat. Studies have shown that patients with *Scedosporium* infection who receive voriconazole have significantly greater survival than those receiving amphotericin B. Further, *S apiospermum* has intrinsic resistance to amphotericin B making voriconazole the first-line treatment [8]. While multiple studies have shown success with voriconazole as monotherapy, there is increasing evidence for the use of a combination of voriconazole and terbinafine [19]. Other successful combinations include triple therapies with voriconazole/caspofungin/amphotericin B [20] and voriconazole/micafungin/granulocyte-macrophage colony-stimulating factor (GM-CSF) [12]. A three-month course of voriconazole was sufficient to treat *Scedosporium* sinusitis in our patient; however, the need for combination therapy in some cases indicates progressive drug resistance. This likely contributes to high mortality in an increasingly immunocompromised population.

This case demonstrates a unique scenario in which *Scedosporium* can cause infection in patients with multiple predisposing factors. Given the increased risk of disseminated infection and mortality in these patients, preventative measures and prophylactic treatments may prove useful.

## Conclusions

This was a case of invasive *Scedosporium* infection in a patient with multiple predisposing factors that was effectively treated with immediate maxillectomy and ethmoidectomy followed by a course of voriconazole. Since *Scedosporium* is a relatively new recognized pathogen causing infection within the last few decades, it is important to understand the varied situations and at-risk populations in which infection can occur. Additionally, immediate and appropriate treatment is of utmost importance given the high mortality associated with localized, CNS, and disseminated infections, especially in immunocompromised patients.
